# The Oxidant–Antioxidant Equilibrium and Inflammatory Process Indicators after an Exercise Test on the AlterG Antigravity Treadmill in Young Amateur Female Athletes

**DOI:** 10.1155/2018/3484159

**Published:** 2018-03-25

**Authors:** Łukasz Sielski, Paweł Sutkowy, Agnieszka Skopowska, Katarzyna Pawlak-Osińska, Zofia Augustyńska, Katarzyna Hewelt, Radosław Drapała, Alina Woźniak

**Affiliations:** ^1^Clinic of Maternal-Fetal Medicine, Gynecology and Neonatology, Ludwik Rydygier Collegium Medicum in Bydgoszcz, Nicolaus Copernicus University, Toruń, Poland; ^2^Multidisciplinary City Hospital, Rehabilitation Center, Bydgoszcz, Poland; ^3^The Chair of Medical Biology, Ludwik Rydygier Collegium Medicum in Bydgoszcz, Nicolaus Copernicus University, Toruń, Poland; ^4^The Chair and Department of Laser Therapy and Physiotherapy, Ludwik Rydygier Collegium Medicum in Bydgoszcz, Nicolaus Copernicus University, Toruń, Poland; ^5^Department of Patophysiology of Hearing and Balance, Ludwik Rydygier Collegium Medicum in Bydgoszcz, Nicolaus Copernicus University, Toruń, Poland

## Abstract

The AlterG antigravity treadmill allows running with a considerable weight reduction. Physical exercise practiced on this treadmill is an innovative method supporting the treatment of injuries in sports and rehabilitation of patients. The aim of the study was to investigate the effect of a 30 min run on the AlterG treadmill with 80% body weight reduction comparing the effect to the similar effort on the classic treadmill on the redox equilibrium and the activity of selected lysosomal enzymes and a serine protease inhibitor in the blood of amateur minor female volleyball players. Venous blood samples were taken before the exercise and 30 minutes and 24 hours after its completion. The obtained results were analysed using Tukey's test and Pearson's linear correlations were calculated. 24 h after the running test on classic treadmill, the erythrocytic superoxide dismutase activity was higher than before and 30 min after it, as well as compared to the run on AlterG treadmill (*p* < 0.001). The erythrocytic-conjugated diene concentration 24 h after the exercise on the classic treadmill was meaningly higher compared to that after the exercise on the AlterG treadmill (*p* < 0.001). The cathepsin D activity was significantly lower after the exercise in AlterG conditions compared to the baseline value and that measured after the exercise on classic treadmill (*p* < 0.001). It seems that the exercise on the AlterG treadmill keeps the oxidant–antioxidant equilibrium and stabilizes lysosomal membranes in young, physically active women in contrast to the exercise on the classic treadmill. This trial is registered with CTRI/2018/01/011344.

## 1. Introduction

New technologies that are currently used in rehabilitation, sports, and long-term care allow a more efficient therapy delivery and are increasingly used in multidisciplinary health care training [1]. One of such innovative methods that support the locomotor capacity is the AlterG treadmill. The concept of this device was created in the US National Aeronautics and Space Administration (NASA). The device was designed to support the physical training of astronauts during their longer stays in space and prevent dystrophies of their skeletal muscles.

The design employed a unique system of air pressure gradient that allows conducting treatment or performing physical exercise in safe conditions, with a partial reduction of load to the musculoskeletal system. It is of particular importance for the exercise of elderly people, athletes, and people with injuries, as load reduction not only allows a quicker restoration of the normal state but also assists or corrects the natural gait pattern. This unique technology minimizes the load and the effects of training that arise during walking and running, as well as enables a gradual return to full fitness. The patient can exercise with the body weight reduced up to 80%. This is due to the significantly reduced force of gravity acting on the musculoskeletal system. An additional purpose for which the AlterG treadmill is used is gait training in patients with neurological conditions. Moreover, in geriatric patients, such exercise allows maintaining physical fitness for longer periods. This new technology is also very helpful in the long process of losing weight and improving health status. The first scientific reports associated with this innovative method come from 2007 and concern a study in a group of athletes after surgical treatment of ruptured Achilles tendon. The study demonstrated that athletes who trained on the AlterG treadmill returned to practising their sports discipline an average of two weeks faster compared to the group that did not practise this form of rehabilitation [2].

Physical effort, especially that of submaximal and maximal intensity, generates load to the body. Depending on the intensity, duration, and repetition, physical effort can be a source of microdamage, as well local inflammation and pain of skeletal muscles [3]. During physical effort, oxygen demand increases, and the body becomes dehydrated and weaker. Body temperature increases significantly, which in extreme cases can lead to heat shock [4, 5]. One of the most important phenomena underlying the changes that accompany physical effort is increased generation of reactive oxygen species (ROS) in the mitochondrial electron transport chain. This results in the intensification of oxidation processes. Oxidative damage of nucleic acids, proteins, and particularly lipids occurs due to the excess of ROS [3]. Among the products of ROS-mediated oxidation process (peroxidation) of lipids are primarily conjugated dienes (CD), as well as aldehydes, including malondialdehyde (MDA)—prevalent among the thiobarbituric acid reactive substances (TBARS) [6]. In this way, lipids that form lysosome membranes can be destroyed [3], which cause the release of lysosomal hydrolytic enzymes. Lysosomes are organelles that respond to rapid disturbances of cell homoeostasis and are involved in the repair of the resulting damage or induction of apoptosis of the cell [7]. Protection from the consequences of excessive ROS generation is provided by heat shock proteins (HSP) and antioxidant enzymes—mainly superoxide dismutase (SOD), catalase (CAT), and glutathione peroxidase (GPx). These antioxidant enzymes are characterized by a high specificity and speed of the catalysed reactions. Their task is to neutralize reactive oxygen species by reducing them [4, 8].

The aim of this study was to investigate the effect of a 30 min run on the AlterG treadmill on the levels of the oxidant–antioxidant equilibrium indicators (CAT, SOD, GPx, CD, and TBARS) and the activity of selected lysosomal hydrolases (acid phosphatase (AcP), arylsulfatase (ASA), cathepsin D (CTS D)), and *α*_1_-antitrypsin (AAT) in the venous blood of amateur minor female volleyball players.

## 2. Material and Methods

The study was approved by the Bioethics Committee of Ludwik Rydygier Collegium Medicum in Bydgoszcz, Nicolaus Copernicus University in Toruń, Poland (consent number: 395/2013), as well as by Clinical Trial Registry-India as clinical study numbered CTRI/2018/01/011344.

### 2.1. Study Subject

Study participants were 27 female volleyball players from an amateur sports club in Bydgoszcz, Poland. The participants had been informed about the course of the experiment and provided their written consent for participation. Their legal guardians were also asked to grant their consent. The participants had been informed about the necessity to keep their dietary and sport habits during the experiment. Characteristics of the study participants were shown in Table 1. Persons after surgical interventions, with chronic diseases and viral and bacterial infections with fever, as well as injuries, were excluded from the experiment.

The experiment was conducted at the beginning of the sports season, when the athletes actively trained for an average of 4 times per week. On a day off training, the athletes underwent a single exercise test (30 min run) on an antigravity treadmill (AlterG; *N* = 18) or on a classic treadmill (*N* = 9) (distance of 5 km at an average speed of 10 km/h). The effort on the antigravity treadmill was performed under the conditions of 80% weight reduction.

The direct experimental material was venous blood taken from the basilic vein into two 4 mL vacuum tubes: for full blood (K_2_EDTA) and blood serum (clot activator and gel separator). Blood samples were taken immediately before the run at rest (measurement 1) and then 30 min (measurement 2) and 24 h (measurement 3) after the exercise completion.

### 2.2. Methods

The measurements were conducted spectrophotometrically. For the assays, erythrocytes, blood plasma, and blood serum were used. The tubes for blood serum were centrifuged for 10 minutes at 1200*g* (+37°C). The serum was the upper layer (supernatant) separated by gel layer. The tubes for blood plasma and erythrocytes were centrifuged for 10 minutes at 6000*g* at +4°C. After centrifugation, the plasma constitutes the upper layer. The lower layer (morphological blood elements) was washed three times with phosphate-buffered saline (PBS), respectively, at a ratio of 1 : 3, and each time centrifuged (the same conditions). Supernatant was deleted to remove leukocytes and thrombocytes. The presence of white blood cells and platelets in the erythrocyte suspension was verified using microscopic observation of the performed smears, whereas the presence of the protein was checked with a 20% aqueous solution of sulfosalicylic acid. Erythrocyte suspension was PBS solution with 50% hematocrit.

In erythrocytes from the blood samples, the activity of the following antioxidant enzymes was determined: CAT (E.C. 1.11.1.6), using the Beers and Sizer method [9]; SOD (E.C. 1.15.1.1), using the Misra and Fridovich method [10]; and GPx (E.C. 1.11.1.9), using the Paglia and Valentine method [11]. The concentrations of CD, following Sergent et al. [12], and TBARS, following Buege and Aust [13], as modified by Esterbauer and Cheeseman [14], were also determined in erythrocytes. The CAT activity was determined by measuring the decrease in the absorbance of a solution of hydrogen peroxide (H_2_O_2_) decomposed by the enzyme [9]. The measurement of the SOD activity was based on the inhibition of adrenaline autoxidation to adrenochrome in alkaline conditions [10]. Determining the GPx activity employed the ability of the enzyme to reduce hydrogen peroxide with a simultaneous oxidation of its cofactor—reduced glutathione (GSH) [11]. The concentration of conjugated dienes in erythrocytes (CDer) was measured by adding chloroform into erythrocyte suspension (1 : 1 erythrocyte in PBS buffer) and collecting the lower fraction of the solution into clean tubes after centrifugation. The samples were subsequently evaporated under nitrogen and dissolved in cyclohexane, and absorbance was read at a wavelength of 233 nm [12]. The concentration of thiobarbituric acid reactive substances in erythrocytes (TBARSer) was determined by adding the following reaction mix to erythrocyte suspension: 0.375% thiobarbituric acid (TBA), 15% trichloroacetic acid (TCA), and 0.25 mol/L HCl. The samples were incubated in water bath for 20 minutes at 100°C, cooled down to 4°C, and centrifuged at the same temperature for 15 minutes. After centrifugation, supernatant was collected, and its extinction was measured at a wavelength of 532 nm [13, 14].

In blood plasma, the following concentrations were determined: conjugated dienes (CDpl) [12], thiobarbituric acid reactive substances (TBARSpl) [13, 14], and vitamins A and E [15]. The concentrations of vitamins were established using HPLC. Denaturation of blood plasma proteins was induced by shaking the investigated solution with 800 *μ*L acetonitrile. After centrifugation, supernatant was filtered, and 4 mL of hexane was added for extraction. The frozen hexane fraction containing vitamins was decanted into clean tubes and evaporated to dryness under nitrogen at 40°C. Then, 100 *μ*L of phase was added to the sample, mixed ultrasonically, and finally injected into an HPLC (C18 column, l = 250 mm) system using a syringe. The detection of vitamins was conducted at the wavelength of 292 nm.

In the blood serum, the activity of the following lysosomal enzymes was determined: AcP (E.C. 3.1.3.2), using the Bessy method, as modified by Krawczyński [9]; ASA (E.C. 3.1.6.1), using the Roy method, as modified by Błeszyński and Działoszyński [16]; and CTS D (E.C. 3.4.4.23), using the Anson method [17]. In the blood serum, the activity of the serine protease inhibitor (AAT) was also determined using the Eriksson method [18]. To assess the activity of AcP, disodium 4-nitrophenylphosphate (substrate) in citrate-tartrate-formaldehyde buffer was used. The measure of enzyme activity was the quantity of 4-nitrophenol (4-NP) released during the enzymatic hydrolysis of the substrate [19]. To determine the activity of ASA, 4-nitrocatechol sulfate (4-NCS) in acetate buffer was used as the enzyme substrate. The measure of enzyme activity was the quantity of 4-nitrocatechol (4-NC) released during the enzymatic hydrolysis of the substrate [16]. The substrate for CTS D was 2% denatured bovine hemoglobin that was dissolved in citrate-phosphate buffer and added to the tested serum. Use of a phenolic reagent generated a blue colour. The intensity of the solution colour was measured spectrophotometrically (*λ* = 660 nm). The concentration of the reaction product was calculated based on the calibration curve for tyrosine (TYR); therefore, the activity of CTS D was expressed in nmol TYR/mg protein/min [17]. The level of the antiproteolytic AAT was determined using the inhibitory effect of this protein on trypsin (TR). The idea was to measure the TR activity after a short incubation with blood serum. The AAT activity was defined in mg of inhibited TR per mL of serum [18].

### 2.3. Statistical Analysis

The obtained results were statistically analysed using a post hoc test (Tukey's HSD test for unequal *N*) of one-way analysis of variance (ANOVA). Pearson's linear correlation coefficient *r* was also calculated for each pair. In post hoc analysis, all assumptions of the ANOVA test (equal size of groups, Levene's test of the homogeneity of variance, and the Kolmogorov–Smirnov test of the normality of distribution) were considered. The results were presented as arithmetic mean ± standard deviation. Differences between the means with *p* < 0.05 were considered to be statistically significant.

## 3. Results

In the study, many statistically significant Pearson's linear correlations were revealed between the assayed parameters in the subjects' blood (Supplementary Table 1 and Supplementary Figure 1 as Supplementary Materials). One of them, correlation between the TBARS concentration and the SOD activity in erythrocytes, was showed graphically (regression analysis, Supplementary Figure 1).

The results are shown in Table 2. Statistical analysis of the results showed that the CDpl concentration measured 24 hours (measurement 3) after the 30 min run on the AlterG antigravity treadmill was higher than that before the effort (measurement 1, difference of 44.4%, *p* < 0.01) and 30 minutes after it (measurement 2, difference of 36.8%, *p* < 0.05). Similar changes were observed in the case of classic treadmill, but the increase was much higher (36% and 240%, resp.). It shows that 30 min after the exercise completion on the type of treadmill, the CDpl concentration was lower compared to the baseline value (*p* < 0.001), what was not revealed after the run on the antigravity treadmill—the CDpl concentrations determined in 1 and 2 measurements were similar (*p* > 0.05). Thus, comparison of both treadmills indicates higher CDpl concentration 30 min after the run on the AlterG treadmill than on the classic treadmill (*p* < 0.05). The CDer concentration 30 min after the AlterG running test was over 5 times higher than that before the effort but only approx. 3 times higher (260%, *p* < 0.001) than that in measurement 3 (24 h posteffort). The opposite tendency was observed for the classic treadmill—30 min after the exercise, the CDer concentration decreased versus the baseline value by 21.7% and meaningfully increased (by 272%) compared to the measurement 24 h after the exercise completion (*p* < 0.001). Assays 24 h postexercise showed that the CDer concentration was higher compared to prerunning value for both treadmills, but in the case of classic treadmill, it was much higher. Differences in CDer concentration between the treadmills are as follows: measurement 1—higher for classic, measurement 2—higher for AlterG, and measurement 3—higher for classic. No statistically significant changes were observed in the concentration of TBARSpl in the subjects similarly in the TBARSer concentration but only in AlterG treadmill conditions. The TBARSer concentration 30 min after the running test on the classic treadmill was higher versus baseline and measurement 3 (24 h postrunning) values (*p* < 0.05 and *p* < 0.001, resp.). However, 24 h after the exercise on the classic treadmill, the TBARSer concentration decreased compared to both prerunning and 30 min postrunning values (*p* < 0.01 and *p* < 0.001, resp.). The TBARSer concentration was statistically significantly higher in classic treadmill conditions compared to that in AlterG excluding measurement 3. Vitamin A concentration was the highest 24 h after the exercise on classic treadmill, and its value was statistically significantly higher compared to the same study timepoint of the AlterG conditions (*p* < 0.05), whereas vitamin E concentration was statistically significant higher in all study timepoints with classic treadmill conditions compared to the AlterG treadmill conditions. Significant increase of the SOD activity was revealed 24 h after the exercise on the classic treadmill compared to measurements 1 and 2, and it was also higher versus the activity measured 24 h after the running test in AlterG conditions (*p* < 0.001). The activities of other antioxidant enzymes, CAT and GPx, changed insignificantly (*p* > 0.05).

The activities of selected lysosomal enzymes in the blood serum of the young sportswomen were similar in both study conditions; nevertheless, a few statistically significant changes were noted. The baseline AcP activity was lower before the run on the classic treadmill than before the run on the AlterG treadmill (*p* < 0.001). In measurement 3 in AlterG results, the enzyme activity decreased compared to the prerunning value (*p* < 0.05). The CTS D activity, which was assayed 30 min and 24 h after the exercise on the AlterG antigravity treadmill, was significantly lower compared to the baseline activity (61.2% and 73.1%, resp., *p* < 0.001) and to the classic treadmill conditions (45.8%, *p* < 0.01, and 60%, *p* < 0.001, resp.). There was no statistically significant changes of the ASA activity, but for both treadmills, the tendency to decrease the activity after the exercise was observed compared to the baseline value (*p* > 0.05). The activity of the serine protease inhibitor, AAT, was different in a statistically significant manner between baseline values only.

## 4. Discussion

### 4.1. Oxidant Stress Indicators

In this study, no statistically significant changes in the activity of antioxidant enzymes in the erythrocytes were observed after the running test on the AlterG treadmill. The CAT activity after the effort slightly increased, while the SOD activity decreased (the changes statistically insignificant, *p* > 0.05). Therefore, the effort did not induce ROS generation in the erythrocytes in the peripheral blood of the amateur female volleyball players subjected to this test. The finding is due to the fact that the O_2_^•−^ radical, generated in oxygen reduction with one electron, is immediately neutralized by SOD [8]. The product of this reaction is another ROS, hydrogen peroxide, reduced in a dismutation reaction by CAT, an enzyme active only at relatively high concentrations of H_2_O_2_, as lower concentrations trigger the response of GPx that shows a greater affinity to the substrate [20]; however, the GPx activity did not change in a statistically significant manner as well. In the posteffort measurements, it was almost identical to the baseline value, at rest, before the effort. However, in the case of similar study protocol, except for treadmill, which was used for the running test, the results were different. Initially, the CAT activity increased 30 min after the exercise on the classic treadmill but then 24 h postexercise decreased, however, in a statistically insignificant manner as well. Nevertheless, the SOD activity 24 h after the exercise was meaningfully higher than before and 30 min after the effort, and it was much higher compared to the result determined 24 h after the exercise on the AlterG treadmill. This is strong evidence on O_2_^•−^ overproduction in erythrocytes 24 h after the run. For the disturbance of the oxidant–antioxidant balance not only in the subjects' erythrocytes but also in blood plasma after the exercise test on the classic treadmill, the results of CD concentrations may also confirm because conjugated dienes are considered as the primary products of the ROS-mediated lipid oxidation [6]. Measurement 2 (30 min postexercise) in comparison to measurement 1 (baseline) showed decrease of the concentrations but compared to measurement 3 (24 h postexercise) showed significant increase. The disturbance could also occur or was initiated in the erythrocytes 30 min after the effort in AlterG conditions, but the balance was restoring, as the CDer level was over 5 times higher 30 min after the exercise and only 2 times higher 24 h afterwards, as compared with the measurement taken immediately before the effort (measurement 1). Relatively low level of exercise-induced ROS generation in the athletes after the effort in AlterG treadmill conditions was also confirmed by statistically insignificant changes in the TBARS concentrations, as among these substances, malondialdehyde prevails (MDA)—a very specific marker and secondary product of lipid peroxidation process, the process induced by ROS [6]. Therefore, statistically insignificant changes of the TBARSpl concentration indicate inconsiderable change in the peroxidation level of the blood plasma or sarcolemma lipids after the effort on AlterG treadmill and TBARSer of erythrocyte membranes [21–23]. In the case of the exercise test on the classic treadmill, the TBARSer concentration in measurements 1 and 2 was higher compared to that in AlterG results, but 24 h after, the test the concentration was already convergent between them (i.e., statistically insignificant, *p* > 0.05). Correlations demonstrated in the study (Supplementary Table 1 and Supplementary Figure 1) show standard interactions between studied parameters and testify the maintaining oxidant-antioxidant balance in the organism; however the reaction can be different, for example, in response to physical effort. In people, which do not perform regular physical training, a significant increase of the SOD activity postexercise is observed, what is rather not observed in professional athletes; however, it depends on the intensity and duration of the effort. Moreover, in professional sportsmen, the activity of this enzyme at rest is higher than that in people who do not practice sports. This is also similar for CAT and GPx activities [24]. Lipid peroxidation products determined in this study (TBARS and CD) are increasingly generated during physical effort, in both amateurs [25] and professionals [26], although in the latter, an opposite reaction of the body can sometimes be observed (plasma and erythrocyte CD↓, plasma TBARS↑) [27], which is explained by adaptation to intense physical effort [3]. Low molecular weight antioxidant levels can be different as well, even in similar subjects. In the study, an increase in the concentrations of exogenous compounds with strong antioxidant properties was observed that deplete during oxidative stress [3]. The concentration of vitamin E was statistically significantly higher in all study timepoints during use of classic treadmill compared to the AlterG results, while vitamin A concentration was only 24 h postexercise. Differences between baseline vitamin concentrations do not correspond with baseline levels of the other parameters (CDer, TBARSer, and AAT). These results are hard to explain but the most likely they could result from differences in dietary habits between both study groups.

### 4.2. Selected Lysosomal Enzymes and Inhibitor of Serine Proteases

For the maintenance of the oxidant–antioxidant equilibrium in the subjects under the single 30 min running test in AlterG treadmill conditions, the results of the activities of lysosomal enzymes may also prove, since one of the mechanisms of release of these hydrolases is peroxidation of the lysosomal membrane lipids due to oxidant stress [3, 7]. Therefore, the demonstrated lack of excessive ROS generation might have directly increased the stability of lysosomal membranes. The activities of AcP and CTS D were statistically significantly lower in the blood serum 24 h after the exercise test on the AlterG treadmill compared to their respective baseline activities (at rest, before the effort), especially the CTS D activity, which was meaningfully lower already 30 minutes after the effort. The CTS D activity after the exercise on the classic treadmill also decreased compared to that on the prerunning activity, but the differences were less and statistically insignificant. The serum ASA activity after the exercise on both treadmills was lower compared to its pre-running activity but statistically insignificant as well. Other authors observed higher activities of AcP and ASA in the blood serum of both professional athletes [28] and amateurs [29] after physical effort. Because the reconstruction of the muscle tissue after physical effort involves neutrophils and monocytes extravasated from peripheral blood, as well as local macrophages [30, 31], these cells at the damaged site release a number of proteins and factors, including proteases [7], lysosomal enzymes [30, 32], and ROS [31]. A link between oxidant stress and the release of lysosomal hydrolases in this study is indicated by the statistically significant correlations observed in the part based on “AlterG protocol.” Before the effort, a negative correlation was observed between the vit. E concentration in the blood plasma and the CTS D activity in the blood serum (*r* = −0.63). Thirty minutes after the exercise test, a positive correlation was demonstrated between the erythrocyte GPx activity and the CTS D activity (*r* = 0.47); however, 24 h after the running test on the AlterG treadmill, the following correlations were noted: the TBARSer concentration versus the AcP activity (*r* = 0.62), the TBARSer concentration versus the CTS D activity (*r* = 0.66), the SOD activity versus the CTS D activity (*r* = 0.53), the GPx versus the AcP activity (*r* = 0.51), the AcP activity versus the vit. A concentration (*r* = −0.49), the CDer concentration versus the ASA activity (0.59), and the CDer concentration versus the CTS D activity (*r* = 0.55). It was also found that the only statistically significant difference in the activity of *α*_1_-antitrypsin (AAT), a serine protease inhibitor and a dominant plasma protein with antiproteolytic properties [33], was noted between its baseline activities in measurement 1, that is, before the exercise test, between the treadmills. The AAT activity was also statistically significantly correlated with other parameters (Supplementary Table 1). Moreover, the AAT activity was higher 30 min after the effort on the AlterG treadmill in a statistically insignificant manner than before its commencement (*p* > 0.05). Twenty-four hours after the run on both treadmills, the AAT activity was also slightly lower than in the measurement taken before the effort (*P* > 0.05). It can therefore be understood that the demonstrated changes in the AAT and the lysosomal enzyme activities probably resulted in the lack of microdamage to the skeletal muscles after the effort especially on the AlterG treadmill in the investigated girls.

## 5. Conclusion

A single 30-min run at an average speed of 10 km/h on the AlterG antigravity treadmill with 80% body weight reduction compared to the exercise with the same duration and intensity but performed on the classic treadmill does not disrupt the oxidant–antioxidant equilibrium and stabilizes lysosomal membranes in young female amateur volleyball players.

## Figures and Tables

**Table 1:** Characteristics of the study participants (mean ± SD).

Characteristic	*N* = 18	*N* = 9
Age (years)	14.5 ± 0.6	15.2 ± 1.0
BM (body mass) (kg)	53.2 ± 6.9	54.2 ± 5.8
BH (body height) (m)	1.7 ± 0.05	1.6 ± 0.03
BMI (body mass index) (kg/m^2^)	19.1 ± 2.3	20.1 ± 1.9
Training experience (years)	4.1 ± 0.3	4.8 ± 0.7

**Table 2:** The levels of primary and secondary lipid peroxidation products, concentration of low molecular weight antioxidants, activities of antioxidant and lysosomal enzymes, and level of serine protease inhibitor in the peripheral blood of young amateur female volleyball players, as a result of a 30-min running test on the AlterG antigravity and classic treadmills.

Study parameter	Measurement 1 (prerunning, baseline value)		Measurement 2 (30 min after running test)		Measurement 3 (24 h after running test)	
	AlterG	Classic	AlterG	Classic	AlterG	Classic
CDpl (10^−2^ Abs./mL plasma)	1.8 ± 0.5	2.5 ± 0.7	1.9 ± 0.4	1.0 ± 0.2^*ααα*†^	2.6 ± 0.7^aab^	3.4 ± 0.9^*αβββ*^
CDer (10^−2^ Abs./g Hb)	1.0 ± 0.3	2.3 ± 0.7^∗^	5.2 ± 1.2^aaa^	1.8 ± 0.5^*ααα*†††^	2.0 ± 0.6^abbb^	4.9 ± 1.5^*αααβββ*‡‡‡^
TBARSpl (10^−1^ nmol MDA/mL plasma)	4.0 ± 0.5	4.6 ± 0.4	4.3 ± 0.6	4.1 ± 0.4	4.4 ± 0.7	4.1 ± 0.3
TBARSer (nmol MDA/g Hb)	19.0 ± 7.0	29.0 ± 3.0^∗∗^	21.5 ± 4.3	37.6 ± 6.9^*α*†††^	18.2 ± 8.0	18.5 ± 3.5^*ααβββ*^
Vitamin A (10^−2^ *μ*g/L plasma)	39.0 ± 7.6	53.5 ± 11.4	48.0 ± 8.5	57.2 ± 11.6	49.0 ± 13.1	65.0 ± 15.0^‡^
Vitamin E (*μ*g/L plasma)	18.9 ± 5.6	29.3 ± 6.0^∗∗^	21.2 ± 5.2	30.4 ± 6.5^†^	22.1 ± 6.9	34.4 ± 5.9^‡‡‡^
CAT (IU/g Hb)	57.5 ± 8.0	63.9 ± 8.4	60.2 ± 8.3	65.2 ± 6.5	63.6 ± 10.8	61.5 ± 6.3
SOD (10 U/g Hb)	74.3 ± 9.6	70.5 ± 7.6	71.8 ± 7.5	65.4 ± 3.2	71.7 ± 6.3	93.5 ± 3.0^*αααβββ*^^‡‡‡^
GPx (U/g Hb)	4.1 ± 2.7	6.4 ± 2.7	4.1 ± 2.6	5.6 ± 2.1	4.8 ± 3.0	5.7 ± 2.6
AcP (10^−4^ nmol 4-NP/mg protein/min)	2.4 ± 0.6	1.3 ± 0.3^∗∗∗^	2.1 ± 0.5	1.5 ± 0.4	1.8 ± 0.7^a^	1.7 ± 0.2
ASA (10^−4^ nmol 4-NC/mg protein/min)	9.3 ± 3.5	7.5 ± 0.7	8.0 ± 2.2	7.0 ± 1.2	7.3 ± 1.9	6.2 ± 0.9
CTS D (10^−2^ nmol TYR/mg protein/min)	13.4 ± 2.9	10.2 ± 1.1	5.2 ± 2.1^aaa^	9.6 ± 0.6^††^	3.6 ± 1.2^aaa^	9.0 ± 1.3^‡‡‡^
AAT (10^−1^ mg TR/mL serum)	7.2 ± 0.8	9.0 ± 1.2^∗^	8.4 ± 1.3	9.0 ± 1.0	7.4 ± 1.6	8.3 ± 1.2

Statistically significant differences compared to the values from AlterG: measurement 1 (^aaa^ *p* < 0.001, ^aa^ *p* < 0.01, and ^a^ *p* < 0.05) and 2 (^b^ *p* < 0.05, ^bbb^ *p* < 0.001) and from classic: measurement 1 (^*α*^ *p* < 0.05, ^*αα*^ *p* < 0.01, and ^*ααα*^ *p* < 0.001) and 2 (^*βββ*^ *p* < 0.001). Comparison between AlterG and classic values was shown as follows: ^∗^ *p* < 0.5, ^∗∗^ *p* < 0.01, and ^∗∗∗^ *p* < 0.001 (measurement 1); ^†^ *p* < 0.05, ^††^ *p* < 0.01, and ^†††^ *p* < 0.001 (measurement 2); ^‡^ *p* < 0.05 and ^‡‡‡^ *p* < 0.001 (measurement 3). CDpl/er: conjugated dienes in plasma/erythrocytes; Abs.: absorbance; TBARSpl/er: thiobarbituric acid reactive substances in plasma/erythrocytes; MDA: malondialdehyde; CAT: catalase; SOD: superoxide dismutase; GPx: glutathione peroxidase; Hb: hemoglobin; AcP: acid phosphatase; 4-NP: 4-nitrophenol; ASA: arylsulfatase; 4-NC: 4-nitrocatechol; CTS D: cathepsin D; TYR: tyrosine; AAT: *α*_1_-antitrypsin; TR: trypsin. Results are shown as mean ± standard deviation.

## Supplementary Materials

Supplementary Materials Supplementary Table 1: Pearson's linear correlations demonstrated between parameters investigated in the study (*p* < 0.05). Supplementary Figure 1: statistically significant Pearson's correlation coefficients (*r*) between the TBARS concentration and the SOD activity in the erythrocytes of the peripheral blood in healthy female amateur volleyball players aged approx. 15 years after the 30 min running test on the antigravity (AlterG) and classic treadmills. TBARS: thiobarbituric acid reactive substances; SOD: superoxide dismutase; MDA: malondialdehyde; Hb: hemoglobin.
