# Supplementary material for: The Oxidant–Antioxidant Equilibrium and Inflammatory Process Indicators after an Exercise Test on the AlterG Antigravity Treadmill in Young Amateur Female Athletes
Source: Oxid Med Cell Longev. 2018 Mar 25;2018:3484159. doi: 10.1155/2018/3484159 (PMC5889893; doi:10.1155/2018/3484159)
Supplement: Supplementary Materials — Supplementary Table 1: Pearson's linear correlations demonstrated between parameters investigated in the study (p < 0.05). Supplementary Figure 1: statistically significant Pearson's correlation coefficients (r) between the TBARS concentration and the SOD activity in the erythrocytes of the peripheral blood in healthy female amateur volleyball players aged approx. 15 years after the 30 min running test on the antigravity (AlterG) and classic treadmills. TBARS: thiobarbituric acid reactive substances; SOD: superoxide dismutase; MDA: malondialdehyde; Hb: hemoglobin. [file 3484159.f1.pdf]

Table 1. Pearson's linear correlations demonstrated between parameters investigated in the study ($p < 0.05$).

Study time point	Correlation (r-Pearson's coefficient)	
	AlterG	Classic
Measurement 1 (before exercise)	Vit. E vs. GPx (-0.51)	TBARSer vs. vit. A (-0.83)
	Vit. E vs. CTS D (-0.63)	CAT vs. vit. E (-0.68)
	TBARSer vs. GPx (0.51)	CDer vs. vit. E (-0.68)
		AcP vs. AAT (0.68)
Measurement 2 (30 min after exercise)	GPx vs. CTS D (0.47)	CAT vs. AAT (0.72)
	AAT vs.CTS D (0.49)	
Measurement 3 (24 h after exercise)	TBARSer vs. vit. A (-0.55)	TBARSer vs. SOD (0.77)
	TBARSer vs. SOD (0.50)	
	TBARSer vs. GPx (0.57)	
	TBARSer vs. AcP (0.62)	
	TBARSer vs. AAT (0.65)	
	TBARSer vs. CTS D (0.66)	
	SOD vs. GPx (0.55)	
	SOD vs. CTS D (0.53)	
	GPx vs. AcP (0.51)	
	AcP vs. vit. A (-0.49)	
	CDer vs. ASA (0.59)	
	CDer vs CTS D (0.55)	

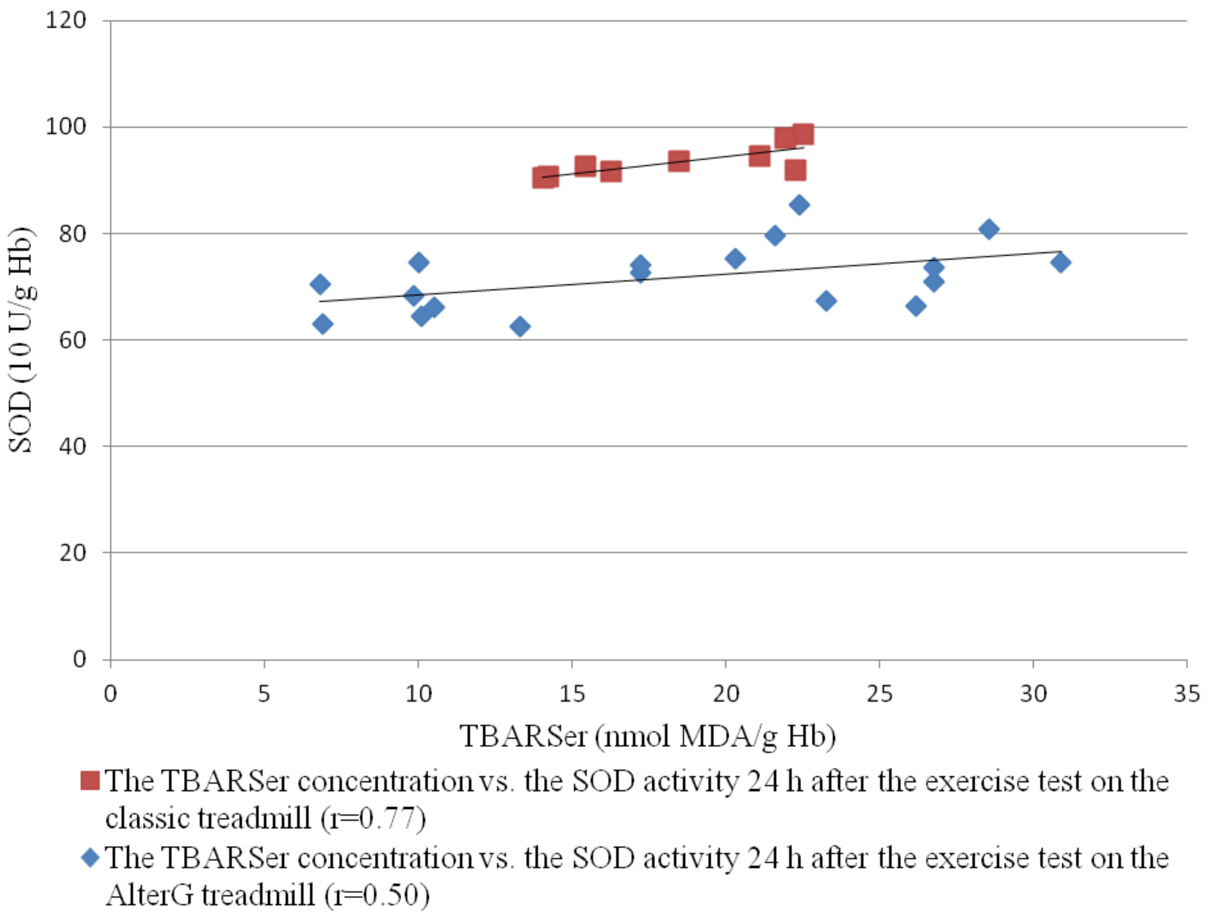

Figure 1. Statistically significant Pearson's correlation coefficients (r) between the TBARS concentration and the SOD activity in erythrocytes of peripheral blood in healthy female amateur volleyball players aged approx. 15 years after the 30-min running test on the anti-gravity (AlterG) and classic treadmills. TBARS, thiobarbituric acid reactive substances; SOD, superoxide dismutase; MDA, malondialdehyde; Hb, hemoglobin.
